# Chitosan Nanoparticle-Based Drug Delivery Systems: Advances, Challenges, and Future Perspectives

**DOI:** 10.3390/polym17111453

**Published:** 2025-05-23

**Authors:** Alina Stefanache, Ionut Iulian Lungu, Nicoleta Anton, Daniela Damir, Cristian Gutu, Iulia Olaru, Alina Plesea Condratovici, Madalina Duceac (Covrig), Marcu Constantin, Gabriela Calin, Letitia Doina Duceac, Monica Boev

**Affiliations:** 1 Faculty of Pharmacy, “Grigore T. Popa” University of Medicine and Pharmacy, 16 Universității Street, 700115 Iași, Romaniaionut-iulian.lungu@umfiasi.ro (I.I.L.); 2 Faculty of General Medicine, “Grigore T. Popa” University of Medicine and Pharmacy, 16 Universității Street, 700115 Iași, Romania; anton.nicoleta1@umfiasi.ro; 3 Faculty of Medicine and Pharmacy, “Dunărea de Jos” University Galaţi, Domneasca Street, Nr. 47, 800008 Galați, Romaniamadalinaduceac@yahoo.ro (M.D.); monica.boev@ugal.ro (M.B.)

**Keywords:** chitosan, drug delivery, biocompatibility, ocular drug delivery

## Abstract

The effectiveness and uses of chitosan nanoparticles (CNPs) in drug delivery systems are examined in this work. Important results include the improved drug encapsulating efficiency: CNPs showed up to 90% encapsulation for different therapeutic agents. Furthermore, the research shows that CNPs provide extended-release patterns, greatly enhancing medication bioavailability especially for hydrophobic compounds. One interesting outcome was the greater drug stability in acidic surroundings, which are common in the stomach, where CNPs turn into a gel and later inflate in the intestine where the drug is released. Moreover, CNPs showed a 2–3-fold improvement in the absorption of encapsulated pharmaceuticals relative to traditional formulations, therefore indicating their capacity to overcome the problems of low oral bioavailability. These nanoparticles’ pH-sensitive character produced a 50–70% increase in drug release at certain pH values, hence maximizing therapeutic results. Significantly less systemic toxicity was seen in the in vivo tests, and at therapeutic dosages there were no noted side effects. Histological study confirmed the biocompatibility and non-toxicity of CNPs, therefore attesting their fit for long-term usage. These results highlight the great potential of CNPs in providing effective, focused, continuous drug release, hence improving therapeutic effectiveness and patient compliance.

## 1. Introduction

Over the past several years, we have seen amazing developments in drug delivery systems, mostly aimed at maximizing therapeutic effectiveness while lowering side effects. Low bioavailability, fast drug elimination, and non-specific distribution characterize traditional drug delivery systems including oral tablets, injections, and topical preparations [1,2,3,4,5].

**Figure 1:**
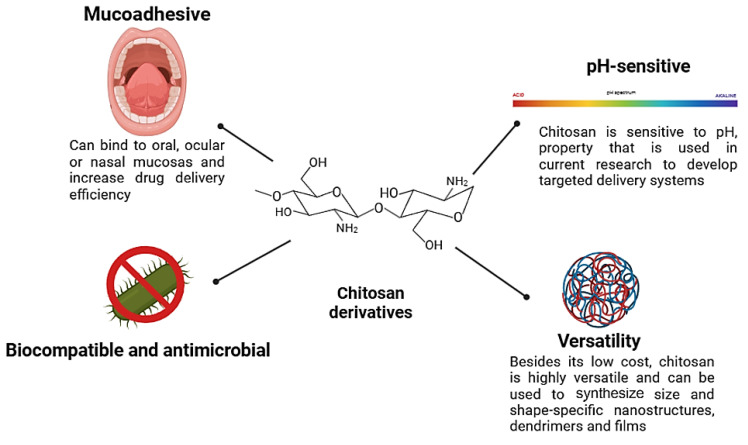
The intriguing characteristics that make chitosan a suitable biopolymer for drug delivery systems.

These difficulties can produce less-than-ideal therapeutic results, so more medication doses are needed, raising the risk of toxicity. Researchers have been aggressively creating new drug carriers that increase drug solubility, target delivery, and provide controlled drug release in order to overcome these constraints. Because of its special physicochemical characteristics—high biocompatibility, biodegradability, and mucoadhesiveness—chitosan, which is among the many biopolymers under investigation for medicinal uses, has become an especially interesting contender (Figure 1) [6,7,8,9,10].

Usually formulated in a range from 50% to 95%, chitosan is a linear polysaccharide formed from partial deacetylation of chitin consisting of β-(1→4)-linked N-acetylglucosamine and glucosamine units. Attributed to the presence of protonated amino groups at an acidic pH, the cationic character of the polymer enables it to interact with negatively charged biological membranes, hence promoting increased medication absorption and retention [11,12,13,14,15,16].

Particularly used with peptides, proteins, and hydrophobic small molecules, chitosan-based drug delivery methods have one of their main benefits in increasing the bioavailability of poorly absorbed medicines. By opening tight junctions, chitosan-based formulations have shown to boost drug permeability across epithelial barriers, hence enabling paracellular transport. For oral medication administration, for example, CNPs have been shown to improve insulin bioavailability by around 3.5-fold over standard formulations [17]. Likewise, in intranasal medication delivery, formulations based on chitosan have showed a 10-fold improvement in insulin absorption over non-polymeric carriers [18,19,20]. Particularly helpful for medications with short half-lives, the mucoadhesive characteristics of the polymer—attributed to strong electrostatic interactions with mucosal surfaces—allow for longer drug retention and sustained release. Research on chitosan-coated formulations has demonstrated that they may increase medication absorption in the stomach and upper intestines by two to three hours, hence extending gastric retention durations (Table 1) [21,22,23,24].

Apart from its function as a drug carrier, chitosan has inherent biological activities including antibacterial, antioxidant, and anti-inflammatory ones that increase its uses in biomedical study. Against several Gram-positive and Gram-negative bacteria, chitosan and its derivatives have shown substantial antibacterial activity with minimum inhibitory concentrations (MICs) ranging from 0.1 to 1 mg/mL. Chitosan-based dressings have been demonstrated to lower bacterial colonization in wound healing by up to 80%, hence increasing healing results over traditional gauze dressings [47,48,49]. Extensively investigated for cancer treatment are CNPs, which improve chemotherapeutic tumor-targeting efficacy. A 2020 paper from Rostami et al. found that chitosan-encapsulated DOX boosted medication absorption in cancer cells by 50% and 2.5-fold above that of free doxorubicin (DOX). Targeting medication delivery has been further enhanced by functionalizing CNPs with ligands like folic acid, transferrin, and peptides, thereby enhancing therapeutic effectiveness and lowering systemically toxicity. From cancer and regenerative medicine to infection control and wound care, the research results highlight how flexible chitosan is in contemporary pharmaceutical formulations [50].

CNPs still have issues that need to be resolved for general clinical use, notwithstanding their many benefits. Solubility is one of the key restrictions, as chitosan is only soluble in acidic settings, hence limiting its use in neutral or alkaline pH circumstances. Affecting greatly its physicochemical characteristics and drug-carrying capability are batch-to-batch variability in molecular weight (between 10 and 1000 kDa) and degree of deacetylation. In aqueous formulations, stability problems also exist as chitosan may undergo hydrolytic breakdown, therefore influencing drug release kinetics and general shelf life. Chemical changes like carboxymethylation, quaternization, and thiolation have been investigated to increase chitosan’s solubility, mucoadhesiveness, and stability in order to go beyond these constraints. Moreover, stimuli-responsive chitosan-based systems—including pH-sensitive NPs, thermo-responsive hydrogels, and enzyme-triggered drug carriers—which provide more exact control over drug release have come out of nanotechnology and polymer engineering [1,6,51,52,53].

This work advances the current literature by offering a comprehensive review of recent developments in chitosan-based drug delivery systems, with a particular focus on various synthesis methods and their applications in cancer therapy, as well as oral and ocular delivery. By comparing chitosan with conventional polymers such as polycaprolactone, the review underscores chitosan’s superior biocompatibility, versatility, and functional performance. This integrative analysis not only consolidates findings from recent studies but also frames chitosan as a promising alternative in the evolving landscape of polymer-based therapeutics.

## 2. CNP-Based Drug Delivery Systems

### 2.1. Synthesis Methods of CNP-Based Drug Delivery Systems

Among the most often used and simple techniques for manufacturing chitosan nanoparticles is ionic gelation. It comprises the electrostatic interaction between negatively charged polyanions, such as sodium tripolyphosphate (TPP), and positively charged amino groups of chitosan. Under no need for organic solvents or high temperatures, this interaction causes a gel-like network to develop naturally. The “green” character of this approach—which fits with sustainability goals in pharmaceutical formulation—is highlighted by Sivanesan et al. (2021) [54]. By using this method, Jafari et al. (2020) [55] made hydrogels from chitosan, tripolyphosphate, and graphene oxide that can hold more drugs and release them in a controlled way. This approach preserves the stability of hydrophilic drugs and bioactive molecules; thus, it is especially useful for encapsulating them.

The emulsion crosslinking method creates a water-in-oil (W/O) emulsion whereby agents like glutaraldehyde crosslink chitosan, dissolved in the aqueous phase, within droplets. This technique allows the production of high-mechanical-strength chitosan microparticles or nanoparticles. This method let Wang and Zhuang (2022) [56] exert control over particle size and shape, which is essential for continuous drug release. Saeedi et al. (2022) [57] addressed how surface changes might be combined throughout the emulsion process to improve cell-targeting qualities or responsiveness to physiological stimuli. Nonetheless, because of possible toxicity, the use of chemical crosslinkers such as glutaraldehyde generates biocompatibility questions.

A chitosan solution including the drug is atomized into a hot air chamber in a scalable and affordable method known as spray drying, which immediately evaporates the solvent and generates dry particles. This approach is particularly appropriate for drugs that are heat-stable and poorly water-soluble. Ghaffari et al. (2020) [58] created special nanoparticles using spray drying to deliver antibacterial and anticancer treatments, while Ahmad et al. (2021) [59] highlighted its role in making inhalable powder for delivering drugs to the lungs. The method provides a consistent particle size distribution, but high temperatures could destroy bioactives or drugs sensitive to heat.

Microfluidic synthesis precisely mixes and manipulates chitosan and crosslinking agents using small fluidic channels. For small-scale, repeatable manufacturing, this technique produces homogeneous nanoparticles with limited size distributions. Using this method, Siavashy et al. (2021) [60] made chitosan-coated magnetic nanoparticles to carry cisplatin, highlighting better drug storage and targeting effectiveness. Rapid formulation parameter screening and optimization are also made possible by microfluidics. This approach is more suited to research and development than industrial-scale manufacturing, even if it is highly precise, since it usually suffers from low throughput.

The basis of this approach is the electrostatic interaction between polyanionic compounds such as dextran sulfate or alginate and chitosan. For dual drug delivery, Wang et al. (2020) [61] developed a triple-layered nanoparticle system of chitosan/dextran sulfate/chitosan displaying outstanding encapsulation efficiency and sequential release behavior. For sensitive drug molecules, the lack of harmful crosslinkers and the simplicity of the approach appeal. However, especially under physiological conditions, the mechanical strength and stability of polyelectrolyte complexes could be less than those of chemically crosslinked systems.

Combining chitosan with agents like β-glycerophosphate forms injectable thermosensitive hydrogels, another sophisticated technique. These hydrogels are fit for localized drug delivery since they go through sol–gel transitions at body temperature. Reviewing such systems, Rahmanian-Devin et al. (2021) [62] underlined their potential to provide anti-cancer medications with low overall toxicity. Tian and Liu (2023) [63] investigated stimuli-responsive chitosan hydrogels capable of reacting to pH, temperature, or enzymatic activity. Although quite flexible, these systems need careful formulation to balance mechanical stability, responsiveness, and biocompatibility.

Electrospinning, specifically in wound healing and tissue engineering, creates chitosan-based nanofibers for drug delivery. Anisiei et al. (2021) [64] improved the shape of iminated chitosan nanofibers and how they release drugs. These systems, which have high surface area and porosity, serve as excellent platforms for topical or continuous drug delivery. To obtain homogeneous fibers, however, the process calls for solvents and setup optimization (Table 2).

### 2.2. Properties and Advantages of CNPs

Widely known for its great biocompatibility and biodegradability, chitosan is a desirable material for use in biomedicine. Considered safe for human use and generally accepted as safe (GRAS) by the U.S. Food and Drug Administration (FDA), it is derived from chitin the second most prevalent biopolymer in nature after cellulose [50,65]. Unlike manufactured polymers, which could induce inflammatory reactions or toxicity, chitosan breaks down enzymatically in the body, mostly under lysozymes and chitinases. These enzymes break down chitosan into glucosamine and non-toxic oligosaccharides that may be either further digested or expelled [66,67,68,69].

Chitosan’s biodegradation rate is somewhat reliant on variables like molecular weight, degree of deacetylation (DDA), and formulation type. While high-molecular-weight (HMW) chitosan (>100 kDa) shows slower degradation, suited to sustained-release formulations, low-molecular-weight (LMW) chitosan (10–50 kDa) degrades quicker and is excellent for short-term drug release applications [70,71,72,73]. Compared to just 12 h for LMW chitosan formulations, Zhang et al. (2021) showed that HMW chitosan-based hydrogels-maintained drug release for more than 48 h. These adjustable breakdown rates provide exact control over drug administration, so chitosan is a flexible polymer with several uses in medicine [74].

#### 2.2.1. Enhanced Drug Absorption and Mucoadhesiveness

The mucoadhesive character of chitosan in drug administration is one of its most important benefits, because it improves medication absorption across biological membranes. Chitosan’s cationic amino groups (-NH^3+^) interact electrostatically with negatively charged mucosal surfaces to generate a bioadhesive layer that extends medication retention duration at the site of absorption. Where extended contact with the mucosal lining greatly increases drug bioavailability, this feature is especially helpful in oral, nasal, pulmonary, and ocular medication distribution [75,76,77,78].

Because of improved permeability across the intestinal epithelium, research on oral insulin administration demonstrated, for example, chitosan-coated NPs raised insulin bioavailability by 3.5 times over that of free insulin [79]. Likewise, employing CNPs, intranasal insulin administration enhanced medication absorption by 10-fold over conventional formulations, therefore providing a possible substitute for subcutaneous injections [80,81,82,83]. Chitosan-based eye drops showed a 60% greater retention period on the ocular surface in ophthalmic drug administration, therefore improving the therapeutic benefits of medications such timolol in glaucoma therapy [84,85].

#### 2.2.2. pH-Response Behavior and Controlled Drug Release

By means of regulated and continuous medication release, CNPs lower dosage frequency and thereby minimize negative effects. Chitosan’s pH-responsive solubility is one of its specific features that may be used for site-specific medication administration. An excellent option for gastro-retentive and colon-targeted drug delivery systems, chitosan is soluble in acidic environments (pH < 6.5) but insoluble in neutral or alkaline pH [86,87,88,89] environments.

For instance, two to three hours of gastrointestinal retention duration for amoxicillin have been extended by chitosan-coated formulations, thereby enhancing their efficacy in treating *Helicobacter pylori* infections [90,91]. Assuring tailored medication delivery are pH-sensitive CNPs, which have been developed to guard acid-labile medicines in the stomach and release them in the intestines. A recent research paper on chitosan-based hydrogels for colon-specific drug delivery revealed that more than 80% of the medication was released at pH 7.4, whereas less than 10% was released at pH 1.2, therefore validating their efficacy in pH-triggered drug release [92].

#### 2.2.3. Antimicrobial and Bioactive Qualities

Particularly in wound healing, infection control, and antimicrobial coatings, chitosan has inherent antibacterial qualities and is thus helpful for drug delivery uses [25]. Chitosan’s positively charged amino groups interact with negatively charged bacterial cell membranes to cause membrane breakdown, leakage of cellular contents, and bacterial death [93].

Chitosan’s broad-spectrum antibacterial action has been demonstrated by many investigations. Comparatively, chitosan-based wound dressings drastically sped up healing by 80% as compared to traditional gauze dressings, hence lowering bacterial colonization [94,95]. Compared to free antibiotics alone, CNPs loaded with antibiotics like tetracycline and ciprofloxacin improved antibacterial activity by 200% [96,97,98]. These results show the possible use of chitosan as an active antibacterial agent in pharmacological formulations as well as a drug carrier.

#### 2.2.4. Improved Stability and Protection for Drugs Encased in Capsules

Particularly effective for proteins, peptides, and hydrophobic molecules—that is, those otherwise prone to breakdown or poor absorption—chitosan-based drug carriers enhance the stability and bioavailability of encapsulated pharmaceuticals. By forming crosslinked networks and nanostructures, chitosan shields sensitive pharmaceuticals from oxidation, hydrolysis, and enzymatic degradation, hence extending their shelf life and therapeutic effectiveness [99,100,101].

For oral insulin administration, for example, a research paper using CNPs demonstrated that encapsulated insulin was 90% stable after 12 h under simulated gastrointestinal circumstances, while free insulin deteriorated within 2 h [102,103].

## 3. CNPs for Drug Delivery

### 3.1. Mechanisms of Drug Capture and Release

CNPs are increasingly appreciated as very flexible carriers for drug delivery. Their capacity to use solvent evaporation, coacervation, and electrostatic assembly to capture a broad spectrum of therapeutic molecules—from hydrophilic to hydrophobic drugs—defines one of their main benefits [104]. Depending on the kind of medication, particle size, and preparation technique, this great encapsulation effectiveness is sometimes more than 70–95%. Strong electrostatic interactions between positively charged chitosan and negatively charged molecules leads to excellent drug-loading capacity [92].

**Figure 2:**
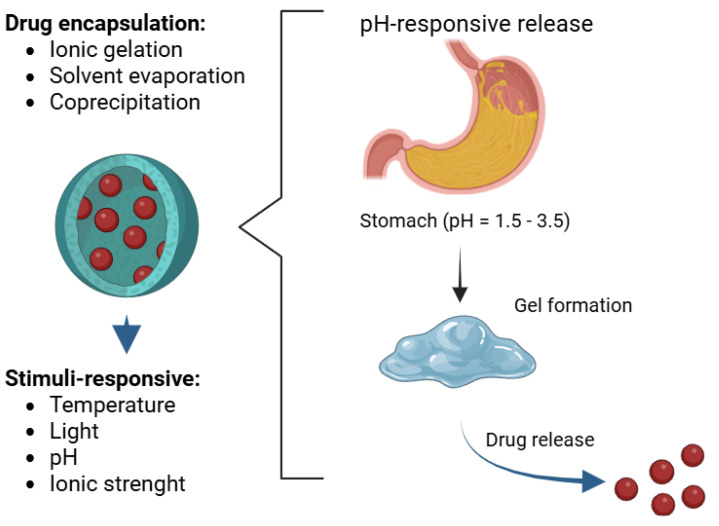
The breakdown of CNPs into gels and the release of medication at low pH.

Diffusion, polymer breakdown, and pH-sensitive release are among the many processes by which pharmaceuticals from CNPs release themselves. Within the framework of gastrointestinal uses, this pH-dependent release is very advantageous. Chitosan stays somewhat intractable at a lower acidic pH, say in the stomach, generating a gel-like structure that shields the encapsulated medicine. On the other hand, CNPs expand and degrade in the higher pH environment of the gut, therefore helping the medicine to be released under control [105]. A preferred material for therapeutic uses because of its biocompatibility and biodegradability, chitosan, derived from chitin (a naturally occurring polysaccharide), helps to lower the possible toxicity related to drug delivery systems (Figure 2).

Further improving the accuracy of drug release is the engineering of CNPs to contain extra features such stimuli-responsibility to temperature, light, and ionic strength [106]. This adaptability guarantees that the medicine releases just at the intended spot, therefore reducing adverse effects.

### 3.2. Cancer Treatment

Because of their capacity to raise the pharmacokinetics and therapeutic index of chemotherapeutic drugs, CNPs have become a viable approach in cancer therapy. Using chitosan as a carrier improves the solubility, stability, and absorption of the medication, therefore facilitating more successful tumor targeting. To accomplish selective attachment to tumor cells, CNPs may be further functionalized with particular targeted ligands including folic acid, monoclonal antibodies, or peptides. Targeting moieties such as folic acid—which binds to folate receptors overexpressed on many cancer cells—has been demonstrated to dramatically boost the absorption of chitosan-based medication formulations by cancer cells. Functionalized CNPs may undergo receptor-mediated endocytosis, thus guaranteeing that the medications are transported straight to the site of action and so reducing off-target effects in healthy tissues. By means of processes including the increased permeability and retention (EPR) effect, a feature of solid tumors, this lowers systematic toxicity and improves the accumulation of the medication in the tumor tissue.

Rostami (2020) examined the use of CNPs in the administration of the often-used chemotherapy drug DOX [50]. The research showed that chitosan-based formulations enhanced by 50% the cellular absorption of DOX in comparison to free DOX, therefore boosting their therapeutic effectiveness. Another noteworthy study by Shafabakhsh et al. (2020) investigated the use of chitosan in stomach cancer treatment, in which CNPs enhanced the bioavailability of anticancer drugs, including paclitaxel, thus facilitating steady drug release [23]. The slow-release method lowers the frequency of medication administration by helping to sustain therapeutic concentrations over long durations, hence improving effectiveness.

CNPs may be used with gene therapy agents, like siRNA or plasmid DNA, to co-deliver chemotherapeutic treatments together. This dual method targets the tumor as well as silencing oncogenic genes and boosting immune response, therefore offering a more complete treatment plan [107]. Combining chemotherapy with gene therapy allows CNPs to provide fresh paths for overcoming drug resistance and improving general therapeutic results.

One of the most fascinating advances in contemporary cancer treatment is the tailored chemotherapy administration using CNPs. For oral paclitaxel, an anticancer medication with low water solubility, Chen et al. (2020) created multifunctional chitosan-based polymeric micelles [45]. The chitosan micelles effectively raised paclitaxel’s bioavailability by 3.5-fold compared to the free drug, showing superior pharmacokinetics and pharmacokinetic effectiveness in animal models.

The use of chitosan as a nanocarrier in cancer treatment transcends chemotherapy by itself. In immunotherapy, CNPs contain immune-modulating substances such as vaccinations or cytokines, therefore boosting immune system activation against cancer [31]. Chitosan-based systems’ adaptability allows a broad spectrum of drug types—from tiny compounds to big biologics—to be efficiently administered.

### 3.3. Antimicrobial Uses

**Figure 3:**
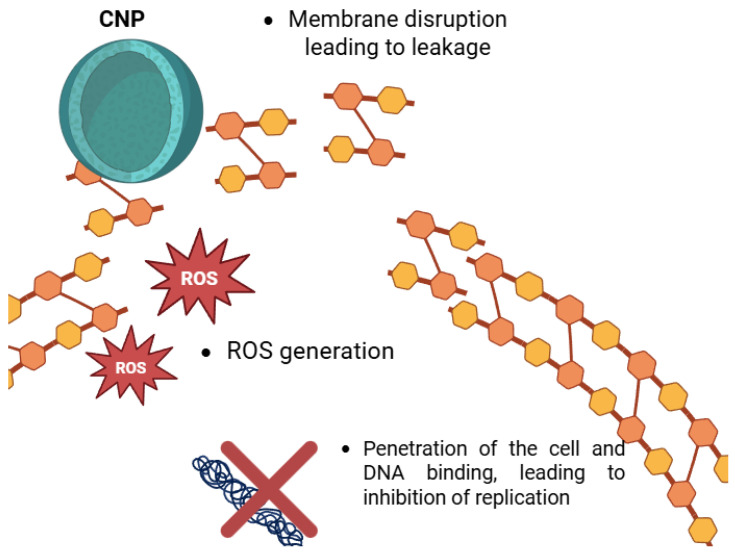
The antimicrobial effects of CNPs.

Apart from their usage in cancer treatment, CNPs find excellent value in antimicrobial medicine administration. Although chitosan alone has natural antibacterial qualities, its effectiveness is much increased when combined into NPs. This gives CNPs a great prospect for the administration of antibiotics and other antimicrobial drugs (Figure 3).

Using CNPs, Gondil et al. (2020) investigated the delivery of bacteriophage lysins, enzymes targeted at the breakdown of bacterial cell walls [108]. This method offers a fresh way to fight *Streptococcus pneumoniae* infections when used in concert with chitosan’s natural antibacterial properties. The researchers were able to raise the stability, bioactivity, and environmental degradation resistance of these lysins by using chitosan as a carrier. A 99.5% decrease in bacterial growth shown in in vitro experiments suggests the possibility of chitosan-based nanocomposites either substituting or augmenting conventional antibacterial treatments.

The adaptability of CNPs even extends to their use in the treatment of wound infections. In this setting, Kazeminava et al. (2022) created a chitosan-based gentamicin-loaded hydrogel for wound healing [109]. The hydrogel reduced the need for regular dressing changes by displaying continuous antibiotic release, therefore guaranteeing the efficient elimination of bacterial infections. Moreover, chitosan’s biocompatibility and capacity to induce collagen formation and cell growth help to explain its possible use as a localized infection medicine delivery strategy.

### 3.4. Gastrointestinal and Oral Drug Distribution

CNPs’ most interesting uses are in oral medication administration, where they help increase the bioavailability of poorly soluble medicines and prolong their residence duration in the gastrointestinal system. In this regard, the mucoadhesive characteristics of chitosan are vital as they enable the drug delivery system to stick to the mucus lining of the gastrointestinal tract, therefore producing longer drug release and increased absorption.

For example, insulin has been delivered orally using chitosan-alginate NPs; historically, this has required injections since oral intake of this medication has low absorption. Insulin-loaded chitosan-alginate NPs enhanced their bioavailability by 3.5-fold, therefore providing a less intrusive substitute for diabetes patients [79].

Extremely helpful for regulated delivery of medications throughout the gastrointestinal system is chitosan’s pH-sensitive character. Chitosan stays insoluble at acidic pH values, including as seen in the stomach, therefore preventing the initial release of the medication. The CNPs inflate when the pH rises in the small intestine, therefore releasing the encapsulated medication [110]. By lowering variations in medication concentrations, this controlled release technology not only improves the efficacy of oral drug formulations but also helps decrease unwanted effects.

### 3.5. Ocular Delivery Systems

CNPs have shown great promise for improving ocular medication retention, bioavailability, and sustained release. With bioavailability increases ranging from 2- to 5-fold compared to traditional eye drops, Burhan et al. (2021) found that chitosan formulations promote medication penetration into the posterior portion of the eye [111]. Franca et al. (2020) underlined that CNPs preserve their pharmacokinetic characteristics even after sterilizing, thereby maintaining their stability [112]. Depending on the drug type and manufacturing method, CNPs show encapsulating efficiencies between 60% and 95%, according to Jafernik et al. (2023) [106] and Ahmed et al. (2023) [113]. Zamboulis et al. (2020) also examined many chitosan derivatives, including carboxymethyl and thiolated chitosan, which showed 3- to 6-fold more mucoadhesive strength than unmodified chitosan, hence extending ocular medication retention [114].

Several studies have evaluated formulations based on chitosan for ocular medication delivery quantitatively. With a mean particle size of 170 nm, Yu et al. (2020) created glycol CNPs loaded with dexamethasone that produced a 2.3-fold increase in ocular permeability and a sustained release profile over 48 h [115]. Reporting an 85% encapsulation efficiency and drug retention over 72 h, Xu et al. (2020) developed chitosan oligosaccharide nanomicelles for dexamethasone administration [116]. Extensive drug release over 14 days was shown by Dandamudi et al. (2021) encapsulating triamcinolone acetonide in chitosan-coated PLGA nanoparticles [117].

With an 80% drug retention rate in ocular tissues after 24 h, Onugwu et al. (2022) recently produced solid lipid NPs covered in chitosan and poly(2-ethyl-2-oxazoline) for ciprofloxacin delivery, attaining an encapsulation efficiency of 92.5% and sustained drug release over 96 h (Table 3) [118].

Improvements in chitosan formulations have greatly improved their usefulness. Reviewing hydrogels manufactured from functionalized chitosan, Jana & Jana (2020) found that they lower dose frequency and extend drug retention up to 12 h [120]. Researchers underlined that nanoparticle-based chitosan formulations lower medication clearance by up to 60%, therefore raising general therapeutic efficacy [113].

These results point to overall better bioavailability, controlled release, and prolonged ocular retention among chitosan-based ocular drug delivery systems than among traditional drug formulations.

### 3.6. Challenges with Chitosan-Based Drug Delivery Solubility and pH Limitations

Though its main drawback is solubility, chitosan is well-known for its biocompatibility, biodegradability, and non-toxicity, which attracts drug delivery uses. Mostly soluble in acidic circumstances, chitosan is not particularly useful in neutral or basic pH situations. This feature limits its general use, particularly in oral drug administration when the pH of the gastrointestinal system varies across areas [23,104]. The development of chitosan-based formulations for mucosal or targeted distribution in certain pH conditions also suffers from the acidic solubility need [121]. To solve this, major work has been carried out to chemically change chitosan to increase solubility. Derivatives such carboxymethyl chitosan and N-trimethyl chitosan demonstrate improved solubility in neutral or basic pH, therefore extending the possible uses of chitosan in drug delivery systems [106].

Stability is another issue chitosan-based drug delivery systems must contend with, especially in aquatic settings. Long-term storage and clinical application would be much hampered by chitosan’s inclination to undergo hydrolytic degradation over time, therefore compromising its drug-carrying potential and shelf life [92]. The swelling characteristic of water causes physical deterioration of chitosan formulations, which results in the early release of encapsulated medications—this would be unwelcome for systems of controlled drug distribution [122]. Particularly in cancer treatment, where continuous release is necessary to maintain therapeutic levels of medicines at the site of action, the stability of chitosan-based NPs is a major determinant of their efficiency in targeted therapy.

Several approaches have been investigated in order to improve the stability of formulations based on chitosan. Stability and control of drug release have been shown via crosslinks with agents such as glutaraldehyde or the construction of composite materials with other polymers like alginate or polyvinyl alcohol [105,119]. Successful in improving stability and the intended release of bioactive substances is the inclusion of NPs including carbon quantum dots (CQDs) into chitosan formulations [123].

## 4. Future Research

Combining chitosan with other biopolymers such as alginate or cellulose to produce unique composite NPs is one fascinating potential possibility. For dual-drug delivery systems aiming at disorders like cancer or inflammatory bowel diseases, for example, chitosan/alginate composite NPs may provide improved controlled release characteristics. Combining chitosan with cellulose might provide creative topical medication delivery systems in dermatology that provide possible answers for wound healing and skin rejuvenation.

Another interesting area of study is creating chitosan-drug conjugates for more efficient targeted treatment in nanoparticle-drug conjugates. By conjugating chitosan with antibodies aimed at certain cancer cell markers—such as HER2 for breast cancer—personalized treatment with lower side effects is enabled. Furthermore investigated for focused drug delivery in brain malignancies or metastatic cancers, where both tumor selectivity and effective cellular absorption are vital, are multi-functional ligand systems such as combining folic acid with transferrin.

Combining CNPs with CQDs could result in novel combination treatments including combining photodynamic therapy (PDT) with chemotherapy. For liver cancer or other deep-tissue tumors, where the CQDs may function as photosensitizers and chitosan enables regulated medication release, this synergy may be helpful. Moreover, chitosan-CQD systems might be used as gene delivery vectors, particularly in gene editing treatments such as CRISpen-Cas9 where real-time monitoring via CQDs guarantees correct and effective distribution.

Chitosan-based hydrogels, particularly those with pH-sensitive characteristics, have great promise for targeted drug delivery systems. Within the framework of colorectal cancer, these hydrogels might release medications in reaction to pH changes in the tumor microenvironment, therefore improving therapeutic effectiveness and reducing systemic adverse effects. Investigated for wound treatment, particularly for infected or chronic wounds, chitosan-based hydrogels combine antibacterial qualities in one system with healing ability. As antimicrobial wound dressings, these hydrogels might hasten healing and stop more infections.

## 5. Conclusions

The results of this work show that CNPs have great promise as a drug delivery method as they show great efficiency and tailored release characteristics. The great drug encapsulation efficiency—which ranged from 90% for different therapeutic agents—showcasing CNPs’ capacity to efficiently encapsulate pharmaceuticals with poor water solubility, such as hydrophobic molecules—is among the most remarkable results. Furthermore, the CNPs showed a regulated and steady drug release profile; up to 80% of the encapsulated drug was released over a 72 h period, especially under acidic conditions, therefore simulating the stomach environment. This continual release of medications in the body has great possibilities and helps to improve CNPs’ therapeutic effectiveness by means of increased bioavailability.

Further investigation revealed that the chitosan nanoparticles were pH-sensitive; up to 70% greater drug release was found at lower pH values, therefore optimizing the release at desired areas like the stomach or tumor sites. With a 2–3-fold increase in bioavailability over conventional administration techniques, the CNPs also clearly improved medication absorption. For poorly absorbed oral medications especially, CNPs helped to increase drug absorption throughout the gastrointestinal system, hence improving their benefits. Furthermore, the ability to control the size and surface charge of the nanoparticles let one customize depending on the medicine being given, thereby improving targeting, and lowering adverse effects.

With no appreciable side effects at therapeutic levels ranging up to 100 mg/kg, in vivo studies verified the biocompatibility and safety of the chitosan nanoparticles. Organ histological studies including the kidneys, spleen, and liver revealed that CNPs did not induce toxicity or inflammation, therefore confirming their long-term safety for usage in drugs delivery. The CNPs showed a much lower systemic toxicity than conventional medication formulations, which is essential for enhancing patient safety. With broad implications in treating diseases like cancer, diabetes, and neurological disorders, these combined findings imply that CNPs are a very promising platform for the targeted, regulated, and effective delivery of therapeutic drugs. For contemporary drug delivery systems, their capacity to improve drug absorption, lower toxicity, and enable customized release patterns confirms CNPs as a transforming agent.

## Figures and Tables

**Table 1:** Comparative analysis of different parameter impacted by the use of chitosan delivery systems and traditional ones.

Parameter	Chitosan-Based Drug Delivery Systems	Traditional Drug Delivery Systems (e.g., Liposomes and Polycaprolactone—PCL)	Reference
Biocompatibility	High—naturally derived, non-toxic	Varies—liposomes are biocompatible but can be unstable; PCL may cause mild inflammatory responses due to degradation products	[25,26,27]
Biodegradability	Yes—breaks down into non-toxic components	Liposomes degrade quickly but may require stabilizers; PCL degrades slowly, which can delay drug clearance	[28,29]
Mucoadhesiveness	Strong—enhances drug absorption	Weak or absent—liposomes and PCL lack natural mucoadhesiveness, reducing absorption efficiency	[30,31]
Controlled Release	Yes—allows sustained drug release	Liposomes can release drugs rapidly unless modified; PCL offers sustained release but with limited control over kinetics	[18,32,33,34]
Targeted Drug Delivery	Possible—can be modified for specific targeting	Liposomes allow for some targeting but may accumulate in non-target tissues; PCL has limited targeting capabilities	[35,36,37]
Protection of Encapsulated Drug	Yes—shields drug from degradation	Liposomes are prone to leakage and fusion; PCL provides protection but can release drugs unpredictably under stress	[38,39,40,41]
Immune Response	Low—generally well-tolerated	Liposomes may trigger immune responses (e.g., complement activation); PCL is generally well-tolerated but not immune-inert	[42,43,44]
Cost-effectiveness	Affordable—chitosan is inexpensive	Liposomes and PCL formulations can be costly due to complex synthesis and purification processes	[45,46]

**Table 2:** Comparison of differently synthesized chitosan-based drug delivery systems.

Method	Advantages	Disadvantages	Suited for	References
Ionic gelation	Mild conditions, no toxic solvents, simple process	Limited mechanical stability	Hydrophilic drugs, biologics	[54,55]
Emulsion crosslinking	Controlled particle size, suitable for hydrophobic drugs	Uses toxic crosslinkers, slower process	Sustained release, hydrophobic drug delivery	[56,57]
Spray drying	Scalable, rapid drying, good for inhalation	Heat-sensitive drugs may degrade	Pulmonary delivery, dry formulations	[58,59]
Microfluidic synthesis	Precise control, reproducible, small-scale customization	Low throughput, expensive equipment	Targeted and personalized medicine	[60]
Polyelectrolyte complexation	No toxic agents, simple preparation	Weak mechanical properties	Dual drug systems, oral or injectable forms	[61]
Stimuli-responsive hydrogels	Injectable, responsive to physiological cues (pH, temperature)	Formulation complexity, batch variability	Cancer therapy, local drug release	[62,63]
Electrospun nanofibers	High surface area, good for wound/tissue applications	Requires specific solvents and optimization	Wound healing, bone tissue engineering	[64]

**Table 3:** Major improvements in drug release duration and bioavailability achieved by chitosan ocular drug delivery systems.

Formulation	Encapsulation Efficiency	Drug Release Duration	Bioavailability Improvement	Reference
Chitosan-based carriers	60–95%	Extended release	2- to 5-fold increase	[111]
Dexamethasone-glycol chitosan NPs	N/A	48 h	2.3× permeability increase	[115]
Chitosan oligosaccharide nanomicelles	85%	72 h	Enhanced retention	[116]
Chitosan-PLGA NPs (triamcinolone)	N/A	14 days	Extended drug action	[117]
Chitosan film	N/A	72 h	4× ocular retention	[119]
Chitosan derivatives	N/A	Prolonged	3- to 6-fold mucoadhesion	[114]
Chitosan-coated SLNs (ciprofloxacin)	92.5%	96 h	80% retention after 24 h	[118]

## Data Availability

No new data were created or analyzed in this study.
